# *Xingnaojing* for Moderate-to-severe Acute ischemic Stroke (XMAS): study protocol for a randomized controlled trial

**DOI:** 10.1186/s13063-017-2222-y

**Published:** 2017-10-16

**Authors:** Xinxing Lai, Kegang Cao, Lingbo Kong, Qiang Liu, Ying Gao, for the XMAS study investigators

**Affiliations:** 1 Department of Neurology, Dongzhimen Hospital affiliated to Beijing University of Chinese Medicine, 5 Haiyuncang, Dongcheng District, Beijing, 100029 China; 2 Institute for Cerebrovascular Disease of Beijing University of Chinese Medicine, 5 Haiyuncang, Dongcheng District, Beijing, 100029 China; 3 Department of Intensive Care Unit, Dongzhimen Hospital affiliated to Beijing University of Chinese Medicine, 5 Haiyuncang, Dongcheng District, Beijing, 100029 China; 4 Center for Evidence-based Medicine, the Word Federation of Chinese Medicine Societies, 19 Xiaoying Road, Chaoyang District, Beijing, 100101 China

**Keywords:** *Xingnaojing* injection, Chinese medicine, Acute ischemic stroke, Randomized controlled trial

## Abstract

**Background:**

*Xingnaojing* injection (XNJ) is widely used for the treatment of stroke in China. However, there is currently a lack of high-quality evidence of its efficacy for acute ischemic stroke. The main objective of this study is to determine whether the addition of XNJ to standard care improves the 3-month functional outcome in patients with acute ischemic stroke (AIS).

**Methods/design:**

The XMAS study is a multicenter, prospective, randomized controlled, open-label trial with a blinded endpoints design. A total of 720 patients will be randomly allocated to either the intervention or the control group in a 1:1 ratio. The intervention group receives XNJ combined with standard care, and the control group receives standard care alone. XNJ will be administered intravenously every 12 h for 10 days. The primary outcome is the proportion of patients who are independent at 3 months after stroke onset defined as a modified Rankin Scale score of 0 to 2. Secondary outcomes include early neurological deterioration at 48 h, the change in National Institutes of Health Stroke Scale score, patient-reported outcome, symptomatic intracranial hemorrhage at 10 days, the Barthel Index score, deaths from any cause and cardiovascular events at 3 months.

**Discussion:**

The results of this trial will provide critical evidence for XNJ in the treatment of AIS as a complementary approach that can be initiated after reperfusion therapy or when the AIS is not eligible for thrombolytic treatment.

**Trial registration:**

Clinical Trials.gov, ID: NCT02728180. Registered on 28 March 2016.

**Electronic supplementary material:**

The online version of this article (doi:10.1186/s13063-017-2222-y) contains supplementary material, which is available to authorized users.

## Background

Globally, stroke is one of the leading causes of death and long-term disability [1]. In the past two decades, the burden of stroke has increased significantly in terms of the absolute number of people with incident stroke, number of deaths, and disability-adjusted life years (DALYs) lost especially in developing countries [2]. According to a nationwide population-based survey in China, there are approximately 2.4 million new strokes and 1.1 million stroke-related deaths annually, with 11.1 million stroke survivors, which consequently results in a huge financial burden [3].

Despite recombinant tissue plasminogen activator (rt-PA) and emerging evidence for endovascular treatment [4], only a few highly selected patients with acute ischemic stroke (AIS) receive thrombolytic therapy due to strict selection criteria. From the Chinese National Stroke Registry, 21.5% of patients with acute stroke presented to the emergency department (ED) within 3 hours, 12.6% were eligible for thrombolytic treatment, and only 1.6% received intravenously administered rt-PA therapy [5]. The lack of effective and widely applicable therapeutic approaches for AIS has resulted in a growing interest in traditional Chinese medicine (TCM).

*Xingnaojing* injection (XNJ) was extracted by modern biotechnology from a well-known Chinese patent medicine named *An-Gong-Niu-Huang Wan* which was first documented in *Wen Bing Tiao Bian*, a classical work of TCM published in 1798. XNJ has been approved by the China Food and Drug Administration (CFDA) and is widely used for the treatment of stroke [6]. XNJ consists of four Chinese herbs: *Moschus*, *Radix curcumae*, *borneol*, and *Fructus gardeniae*. Previous studies have demonstrated that XNJ may has multiple neuroprotective mechanisms in terms of inhibiting glutamate-induced apoptosis [7], anti-autophagy via the p53-DRAM signaling pathway [8], decreasing gamma-aminobutyric acid (GABA) [9], improving neurobehavioral disturbances, as well as reducing infarct size [10]. Recently, a systematic review and meta-analysis to assess the efficacy of XNJ for stroke demonstrated that XNJ may reduce brain injury and improve functional recovery after stroke. Due to methodological limitations of the included trials, however, the efficacy of XNJ for ischemic stroke is still not established [11].

Thus, in the present study, we aim to determine whether the addition of XNJ to standard care, intravenously administered within 24 h of symptom onset, improves the 3-month functional outcome in participants with AIS.

## Methods/design

### Study design

The *Xingnaojing* for Moderate-to-severe Acute ischemic Stroke (XMAS) trial is a multicenter, prospective, randomized controlled, open-label trial with blinded endpoints assessment (PROBE design). We compare XNJ, in combination with guideline-based standard care, versus standard care alone in patients with acute ischemic stroke within 24 h after stroke onset. A completed Standard Protocol items: Recommendations for Interventional Trials (SPIRIT) Checklist for the trial is available in Additional file 1.

### Recruitment

**Fig. 1:**
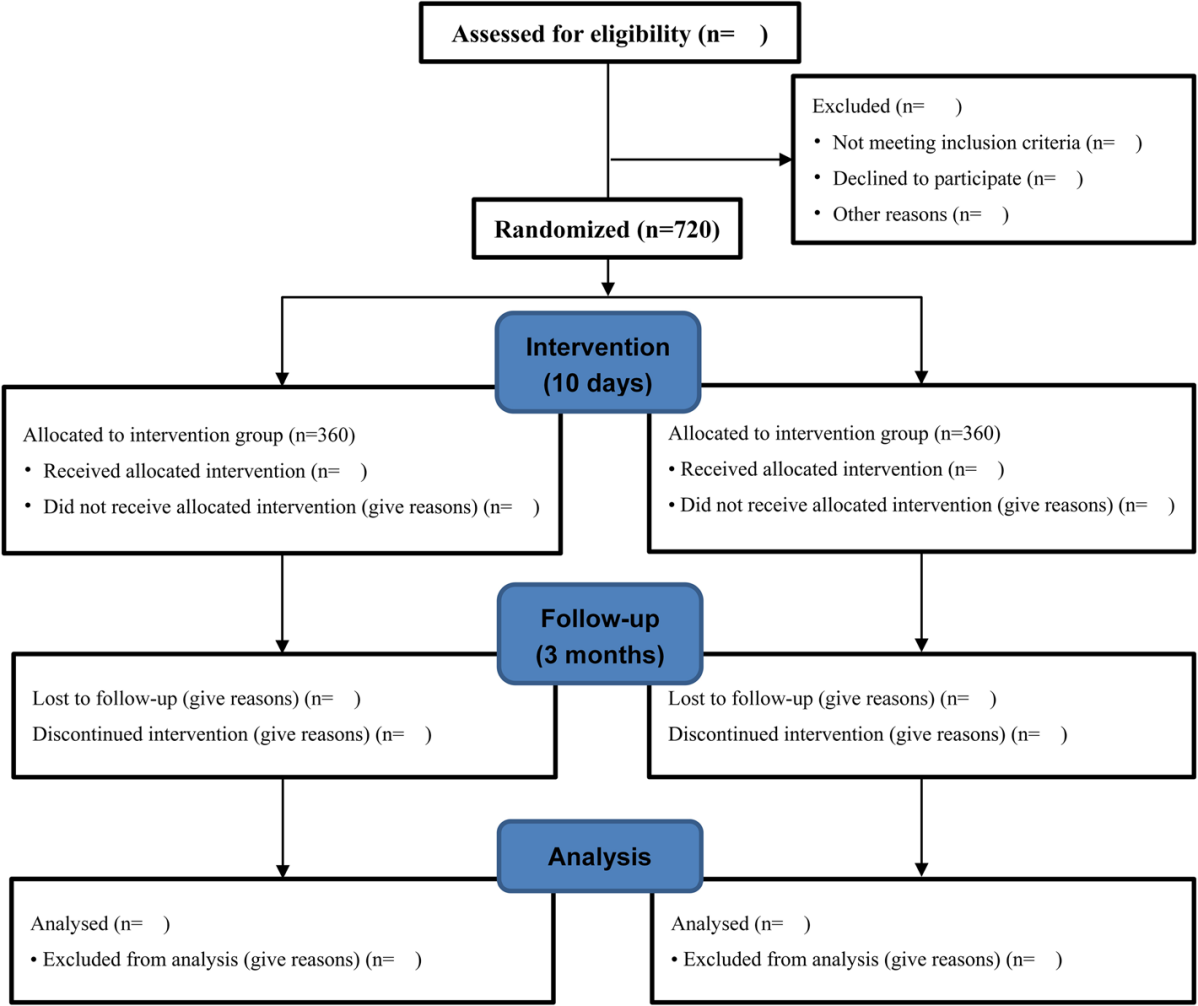
Flow diagram of the *Xingnaojing* for Moderate-to-severe Acute ischemic Stroke (XMAS) study

Patients will be recruited from 30 tertiary public hospitals throughout China with EDs and neurology wards that receive patients with AIS. The recruitment started in March 2016 and is estimated to end in January 2018. Patients with moderate-to-severe AIS within 24 h after stroke onset will be screened for eligibility according to the inclusion and exclusion criteria. Moderate-to-severe was defined as the National Institutes of Health Stroke Scale (NIHSS) scores ranging from 5 to 20. Time of stroke onset is defined as the last time when the patient was witnessed to be well. Those who have stroke symptoms upon awakening (wake-up stroke) will be considered to have their onset at beginning of sleep. Only those who meet the inclusion criteria and willingly provide written informed consent will be included. The flow diagram of this study is shown in Fig. 1.

### Inclusion criteria

- Acute ischemic stroke within 24 h of symptom onset
- NIHSS score ≥ 5 and ≤ 20
- Age ≥ 35 and ≤ 80 years
- Patient or legally authorized representative has signed informed consent

### Exclusion criteria

- Planned or already receiving endovascular treatment
- Suspected secondary stroke caused by tumor, brain trauma, or hematological diseases
- Already dependent in activities of daily living before the present acute stroke (defined as modified Rankin Scale score ≥ 2)
- Other conditions that lead to motor dysfunction (e.g., severe osteoarthrosis, rheumatoid arthritis)
- Significant renal or hepatic insufficiency (defined as a serum creatinine concentration, alanine aminotransferase (ALT), or aspartate aminotransferase (AST) value that is twice the upper limit of normal)
- Life expectancy of 3 months or less due to other life-threatening illness (e.g., advanced cancer)
- Other conditions that render outcomes or follow-up unlikely to be assessed
- Known to be pregnant or breastfeeding
- Currently receiving an investigational drug

### Randomization

All participants will be randomly allocated to the intervention group or the control group in a 1:1 ratio. The randomization procedure will be computer- and web-based, using permuted blocks, and will be stratified for medical center. The randomization algorithm for the study will be generated and uploaded to the randomization system before recruitment. It will not be possible to know the treatment allocation before randomization. The password-protected randomization system requests a few key items of baseline data including the time of stroke onset, NIHSS score, date of birth, as well as a double check on inclusion and exclusion criteria, which are then entered with the computer keypad or tablet computer. When the data have been entered and checked, the computer will generate the treatment allocation. Participants who do not meet the criteria will be excluded by the system automatically and will not be randomized.

### Blinding

In this PROBE design trial, both participants and treating clinicians will be aware of the treatment assignment. Information including NIHSS score, Glasgow Coma Scale (GCS) score, and Patient-reported Outcome (PRO) score will be assessed by independent assessors who are blind to the assignment and treatment. Patients will be contacted over the telephone by independent trained interviewers, who will use standard structured scripts to collect planed data at 30 days and 3 months after stroke onset. The principal investigator, statistician, and outcomes assessors were unaware of the treatment assignments throughout the trial until the database was locked.

### Interventions

Patients randomly assigned to the intervention group will receive XNJ injection, which will be manufactured by the Jiminkexin Group, combined with standard stroke care. XNJ will be administered intravenously in the ED or neurology ward immediately after completion of the randomization. The infusion will contain 20 ml XNJ diluted in 250 ml normal saline, infused every 12 h for 10 days. All information regarding XNJ administered to each participant will be recorded by nurses including dosage, administration time, and details of discontinued treatment.

Those who are assigned to the control group will be given standard stroke care alone. The standard stroke care follows the current national guidelines for acute ischemic stroke from the Chinese Society of Neurology, including anti-platelet therapy, control of vascular risk factors, and appropriate rehabilitation, which will be determined by the attending physician. Treatment with tissue plasminogen activator (rt-PA) within 3 to 4.5 h of symptom onset, after exclusion of intracranial hemorrhage, is encouraged in participants who meet thrombolytic treatment criteria outlined in guidelines.

Neither traditional Chinese herbal medication nor acupuncture is allowed during the hospitalization. All concomitant medications will be recorded in the Case Report Forms (CRFs) including the name, dosage, and course of these medications.

Participants may withdraw at any time during the trial. Investigators will discontinue the treatment in the following cases: the occurrence of serious adverse events; at the request of participant or legally authorized representative to discontinue; and other reasons for consideration of safety. All reasons of discontinuing will be recorded in the CRFs.

### Outcome measures

#### Primary outcome

The primary outcome is the proportion of patients independent at 3 months after stroke onset defined by a mRS score of 0, 1, or 2. Scores on this scale range from 0 to 6, with higher scores indicating greater disability [12].

### Secondary outcomes

- Comparison of the change of degree of neurological deficit from baseline to day 10 or discharge, according to the NIHSS scores ranging from 0 to 42, with higher scores indicating more severe neurological deficits [13]
- Comparison of the PRO scale of stroke at 10 days, which consists of four dimensions including the influence on physical, emotional, and social functioning, as well as the overall satisfaction with treatment [14]
- The proportion of patients with a Barthel Index (BI) score of ≥ 90 at 30 days and 3 months after stroke onset. The BI is used to evaluate the activities of daily living, ranging from 0 to 100, with higher scores indicating more independence [15]
- The proportion of early neurological deterioration (END), defined as an increase of 3 points or more in the NIHSS score between baseline and 48 h
- The proportion of symptomatic intracranial hemorrhage (sICH) occurrence within 10 days of stroke onset, defined as any ICH related to a decline in neurological status or the development of new neurological symptoms in the judgment of the clinical investigator
- The incidence of cardiovascular events, which consist of recurrence of stroke or myocardial infarction
- The incidence of deaths from any cause within 10 days and 3 months after symptom onset
- Safety endpoints, which consist of any adverse events, results of electrocardiography, vital signs and laboratory tests (complete blood count, chemistry, and urinalysis)

### Safety reporting

Adverse events (AEs) are defined as any unfavorable and unintended sign, symptom or disease arising in participants in both groups during the trial period, whether or not related to the experimental treatment. The investigator should take appropriate measures to ensure the safety of the participants and follow up the outcome of any AEs (clinical signs, laboratory values or other, etc.) until the return to normal or stabilization of the patient’s condition. All AEs reported by the subjects, caregivers, or observed by the treating physicians during this trial will be recorded, including the nature of each event, onset date and time, duration, intensity, assessment of its cause, specific therapy and its outcome.

A serious adverse event (SAE) is one that can fatal or life-threatening, requires prolonged hospitalization, or results in permanent or substantial disability. In case of SAEs, the principal investigator will be notified by email or telephone within 24 h. The principal investigator subsequently reports a SAE to the Data Safety Monitoring Board (DSMB). This is an independent committee of trial experts that will focus on safety monitoring to ensure that the study meets the highest standards of ethics and patient safety.

### Data collection

An overall schedule of trial-related activities and data collection of this study is shown in Table 1. All data will be collected on standardized CRFs designed for this study.

**Table 1:**
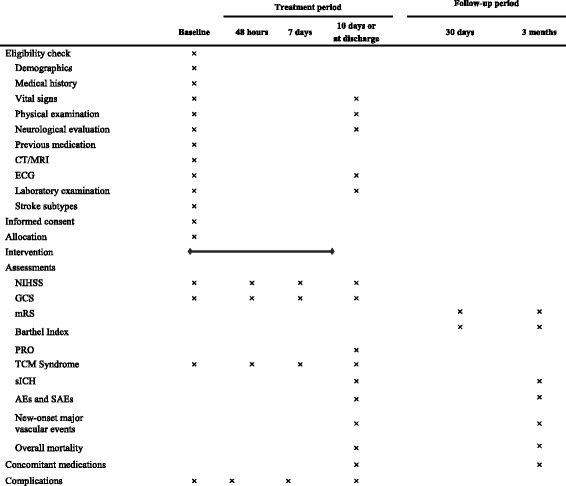
Schedule of trial-related activities

Abbreviations: *CT* computed tomography scan, *MRI* magnetic resonance imaging, *ECG* electrocardiogram, *NIHSS* National Institutes of Health Stroke Scale, *GCS* Glasgow Coma Scale, *mRS* modified Rankin Scale, *PRO* Patient-reported Outcome Scale of Stroke, *TCM* traditional Chinese medicine, *sICH* symptomatic intracranial hemorrhage, *AEs* adverse events, *SAEs* serious adverse events

Baseline characteristics are collected including demographics, medical history, previous medication, pre-stroke mRS, vital signs, GCS score, NIHSS score, Diagnostic Scale of Syndrome Elements in Ischemic Stroke (DSSEIS) score, time of symptom onset, time of ED arrival, time of intervention, stroke subtypes, and complications.

Independent assessors at each center, who were trained before enrollment, will be responsible for assessment including NIHSS score, GCS score, DSSEIS score, and PRO score at 48 h, 7 days, and 10 days or at discharge if earlier.

In addition, CD-ROM copies of all computed tomography (CT) and magnetic resonance imaging (MRI) scans will be collected from participating centers and assessed blindly by independent neuro-radiologists at the central trial imaging analysis center at the Institute for Cerebrovascular Disease of Beijing University of Chinese Medicine (ICVD).

### Follow-up

Independent interviewers will contact all subjects by telephone and collect follow-up information using standard Telephone Interview Forms (TIFs) at 30 days and 3 months after stroke onset. The TIF is a standard questionnaire including structured mRS, BI, locations, stroke recurrences, new-onset vascular events, self-reported information on medication use, and rehabilitation at follow-up period. All telephone interviewers, with a neurological background, will be trained to contact participants according to a standard operating procedure (SOP) before trial recruitment. All interviews will be recorded and retained.

### Data management and quality assurance

To ensure the accuracy and reliability of the data, an independent organization will be responsible for the data management. After the first participant has completed the study at each site, a site visit will be performed by the trial monitor. The monitor will review the CRFs and compare to the patient’s source medical record. Any inadequacies or errors will be reviewed with the local co-investigator and coordinator. Subsequently, site monitoring visits will be performed after every six participants enrolled at that site.

Data entry from the CRFs and TIFs will be performed in the web-based system by trained data-entry personnel using independent, dual data entry. Data are entered twice with each entry performed by a different personnel. Data-entry operators are trained to enter exactly what is recorded on the CRFs. Every structured field that is entered into the database has either a range check or internal consistency check, or both. Data-quality checks are performed by the clinical data system after the independent, dual data entry. All discrepant entries will be marked and recorded for resolution at the clinical data system. Subsequently, a trial monitor will review the two entries, resolve discrepancies, and query the entry operators and local co-investigator if necessary. All discrepancies are manually reviewed.

All study-related data and documentation will be retained by the Data Management Center at the Institute for Cerebrovascular Disease of Beijing University of Chinese Medicine (ICVD).

Several approaches were used to coordinate the different centers in order to avoid the potential heterogeneity of data. Firstly, investigators from all centers were trained to understand the clinical trial protocol and were required to comply with the standard procedure through centralized and site training before recruitment. Secondly, all assessors at each center and telephone interviewers were trained before enrollment to assure consistency among assessors. Thirdly, an independent monitoring team will be responsible for reviewing the CRFs and make sure that they comply with the medical record.

### Sample size

Based on the Third International Stroke Trial (IST-3), it is expected that 35% of the patients with AIS treated with usual care will have an independent outcome (mRS 0–2) [16]. According to previous studies [17–19], the absolute increase in the proportion of patients independent varied from 12 to 27%. Conservatively, a 12% absolute increase in the intervention group is assumed, compared with the control group. With a power of 85% and a two-sided test of a 5% type I error, a sample size of 594 would be needed. Considering the dropout rate, approximately 720 participants will be enrolled in this study.

### Statistical analyses

Statistical analysis will be executed on the intention-to-treat principle. All statistical tests will be performed at the 0.05 level of significance, unless otherwise specified. The Statistical Analysis System (Version 9.2, SAS Institute Inc., Cary, NC, USA) will be used to perform the statistical analyses.

Baseline characteristics will be summarized by means of simple descriptive statistics and be compared between the two treatment groups to assess covariate balance. The two-sample Student’s *t* test will be used for continuous variables and the chi-square test or Wilcoxon test will be used for categorical variables.

The primary analysis will be a comparison of the proportion of patients in each group who are independent (mRS 0, 1, and 2) at 3 months, for all those allocated XNJ versus all those allocated control using Pearson’s chi-squared test. Additional analysis will performed using the generalized Cochran-Mantel-Haenszel (CMH) test statistic to compare the distribution of mRS scores between groups.

Secondary analyses include the reduction of NIHSS, the total score and score in four dimensions of PRO, incidence of cardiovascular events, mortality, the proportion of BI scores ≥ 90, END, and sICH. Secondary outcome analyses will be carried out according to standard statistical principles for comparison of parametric or non-parametric distributions as appropriate. Multivariable regression analysis will be used to adjust for chance imbalances in the main prognostic variables between groups, such as age, stroke severity (NIHSS score), time since onset, ischemic stroke subtype, whether atrial fibrillation, and whether diabetes mellitus.

Pre-defined subgroup analyses will be performed of the effect of treatment at 3 months, subdivided by the following baseline features: time since onset, age, gender, clinical stroke syndrome using the Trial of Org 10172 in Acute Stroke Treatment (TOAST) classification, stroke severity according to the NIHSS, blood pressure at randomization, and TCM syndrome (Zheng, a key concept in TCM) [20].

### Missing data

Missing data may cause seriously compromised inferences to scientific credibility of causal conclusions from clinical trials. Missing data are mainly caused by discontinued treatment due to adverse events, lack of tolerability, lack of efficacy, or simple inconvenience. To limit the extent of missing data, several approaches are taken according to previous experience [21]. When the assigned treatment is discontinued, efforts will be made to obtain the participant’s consent for the collection of data on treatments and outcomes. Patients with no data available after randomization will have worst-case values assigned for the 3-month data point (e.g., mRS = 6, BI = 0).

## Discussion

In the XMAS study with a multicenter PROBE design, placebo is not used as the control group. A double-blind, placebo-controlled designed trial might be optimal; however, this design would lead to considerably higher costs as compared with the PROBE design. A potential advantage of the PROBE design is that the effect of XNJ in our trial will resemble the effect in clinical practice. The PROBE design has been widely discussed and demonstrated to yield the same result as a placebo-controlled design [22, 23]. Moreover, the PROBE design has been performed in several previous studies of stroke intervention [16, 24].

Our study has several limitations. First, since this study is undertaken in China, it is uncertain whether the effects of XNJ would be similar in other ethnic groups. Moreover, due to the relatively short follow-up period of 3 months, it will remain unknown whether XNJ can reduce vascular events and mortality over longer periods.

In summary, the aim of the XMAS study is to determine whether the addition of XNJ to guideline-based standard care increases the proportion of patients independent at 3 months after AIS. If positive, this study will provide critical evidence for XNJ, a traditional Chinese patent medicine, as a complementary approach that can be initiated after reperfusion therapy or when the AIS is not eligible for thrombolytic treatment.

### Trial status

Recruitment to the study started in June 2016. The trial is currently on going.

## Additional file

Additional file 1: The SPIRIT Checklist. (DOC 127 kb)

## Abbreviations

AEs: Adverse events
AIS: Acute ischemic stroke
ALT: Alanine aminotransferase
AST: Aspartate aminotransferase
BI: Barthel Index
CFDA: China Food and Drug Administration
CM: Chinese medicine
CRFs: Case Report Forms
CT: Computed tomography
DALYs: Disability-adjusted life years
DSMB: Data Safety and Monitoring Board
DSSEIS: Diagnostic Scale of Syndrome Elements in Ischemic Stroke
ED: Emergency department
END: Early neurological deterioration
GCS: Glasgow Coma Scale
ICH: Intracranial hemorrhage
ICVD: Institute for Cerebrovascular Disease of Beijing University of Chinese Medicine
IRB: Institutional Review Board
IST-3: The Third International Stroke Trial
ITT: Intention-to-treat
MRI: Magnetic resonance imaging
mRS: modified Rankin Scale
NIHSS: National Institutes of Health Stroke Scale
OCSP: Oxfordshire Community Stroke Project
PRO: Patient-reported outcome
PROBE: Prospective, randomized controlled, open-label trial with blinded endpoints assessment
RCT: Randomized controlled trial
rt-PA: Recombinant tissue plasminogen activator
SAEs: Serious adverse events
TCM: Traditional Chinese medicine
TIFs: Telephone Interview Forms
TOAST: Trial of Org 10172 in Acute Stroke Treatment
XNJ: *Xingnaojing* injection
